# Dynamic relationship of traditional soil restoration practices and climate change adaptation in semi-arid Niger

**DOI:** 10.1016/j.heliyon.2020.e03265

**Published:** 2020-01-18

**Authors:** Abdourhimou Amadou Issoufou, Idrissa Soumana, Garba Maman, Souleymane Konate, Ali Mahamane

**Affiliations:** a WASCAL Graduate Research Program on Climate Change and Biodiversity, Université Félix Houphouet Boigny, Cote d'Ivoire; b Institut National de la Recherche Agronomique du Niger, Niger; c Université Nangui Abrogoua, Cote d'Ivoire; d Univesité de Diffa BP78 Diffa, Niger

**Keywords:** Agricultural science, Environmental science, Scholars and local knowledge, Southwest Niger, Climate change adaptation, Soil restoration strategies, AquaCrop model

## Abstract

Climate change increases the vulnerability of agrosystems to soil degradation and reduces the effectiveness of traditional soil restoration options. The implementation of some practices need to be readjusted due to steadily increasing temperature and lowering precipitation. For farmers, the best practice found, should have the potential to achieve maximum sustainable levels of soil productivity in the context of climate change. A study was conducted in South-West Niger to investigate the use of the suitable practice, through (i) a meta-analysis of case studies, (ii) using field survey and (iii) by using AquaCrop model. Results showed that the effects of the association zaï + mulch on crop yield was up to 2 times higher than control plots depending on climate projections scenario RCP 8.5 under which carbon dioxide (CO_2_) concentrations are projected to reach 936 ppm by 2100. The practice appeared to be an interesting option for enhancing crop productivity in a context of climate change. Concerning its ability, it offers the best prospects to reverse soil degradation in the study area. In addition, the simulation showed that this strategy was suitable for timely sowing and therefore confirmed scholars and farmers views. Furthermore, this practice is relatively more effective compared to the others practices. These results show that association zaï + mulch could be considered as the best practice that can participate to a successful adaptation to reduce risk from climate change at the same time by reducing the vulnerability of farmers in Southwest of Niger for now and even for the future.

## 1 Introduction

Soil degradation and Climate change hinder agricultural productivity and the ability of the agricultural sector to feed the World's increasing population [1]. This issue is of particularly importance in Niger since 1970s. In this country, agriculture sector is characterized by arable land scarcity and highly variable rainfall. Despite plowing is the most soil management practice, farmers saw how in such an unforgiving and harsh climate, that plowing is not effective [2]. Average temperature has increased over most of West Africa in the latest century and is looked for going on during this century and after [3]. Tendency in precipitation are projected to go up and decline in the Sahelian countries such as Niger [4]. Such changes in weather patterns will likely affect crop yield and restoration outcomes and make it difficult to meet the sustainable development goals (SDGs) such as zero hunger and climate action [5, 6]. But, not all effects of climate change may be adverse to agronomic or food production. Certainly, there will also be favorable effects in some regions [7]. Africa appears ill prepared to adapt to or mitigate the powerful effects of climate change. With no adaptation strategies in place, by the year 2020, 75–250 million people in Africa will be exposed to high water stress conditions with some countries experiencing up to a 50% reduction in yields from rain-fed agriculture [8].

There are several technical solutions to soil fertility restoration, many with similar fundamental principles, but their successes depend upon practical relevance, efficiency of application, and acceptance by the farmer. The major sets of benefits from sustainable soil restoration practices are: (i) higher stable production output; (ii) adaptation to climate change and reduced vulnerability; (iii) enhanced ecosystem functioning and services. This is especially important for the local communities in Sahelian countries who depend completely on rain-fed agriculture [9]. Such communities, are already struggling to manage efficiently with the impacts of current climate variability. But suitable traditional soil restoration may maintain or even improve the ability of agroecosystems to supply soil functions to face the future climate change. Considering uncertainty about future climate change, communities may take no-regrets responses options that are favorable in addressing current management regardless of whether or how climate may change in the coming time. Identifying systems of maintaining or improving agronomic productivity, without degrading soil fertility, 9.2 billion people by 2050 can be fed with judicious/prudent diet while also restoring soil and water and mitigating the climate change [7].

Determining the effectiveness or robustness of current soil restoration practices under changing climatic conditions that are supposed to prevail in the future is sought by this study. The objectives of this study were therefore to address the following questions. First, what are the different soil restoration practices implemented in the southwest of Niger? Second, which practice fit better with the ongoing climate change and the future climate? Finally, how best restoration practice efficiency will be affected by climate change in the study area?

## 2 Method

### 2.1 Study area

The study was carried out in the southwest of Niger particularly in Simiri, Tabla and Kollo, sahelian agro-pastoral zone where millet (Pennisetum glaucum) is the predominant annual crop. Rainfall pattern is modal with a long dry season lasting from October to May and a short rain season from late June to September, hence one harvesting period per year. The annual mean rainfall over the last decade in the region is 380 ± 135 mm over a rainy season of 4 months, with a downward trend. The region has a mean annual of temperature range from 36.5 °C accompanied by important evapo-transpiration rates. The climate is however unforeseeable, extreme droughts continue for several years and some rainy seasons fail completely [10]. The vegetation is mostly dominated by annual grasses and bushes and its dynamics are highly adapted to droughts. The soils of the region are arenosols characterized by inherent low soil fertility, poorly developed soil structure and limited organic matter content [11]. Soil nutrient deficiency is a limiting factor in assessing the productiveness. Meanwhile, agriculture has an important value in local population's livelihood. The majority of farmers in the study area implement soil restoration practices and therefore, have built a long experience in restoring such marginal lands.

### 2.2 Analysis of traditional soil restoration practices

#### 2.2.1 Study selection

We investigated published peer-reviewed journal articles that evaluated the response of crop yield response to climate change, using a meta-analysis of impacts of soil restoration strategies on soil productivity in a context of climate change. To minimize publication bias, only studies that satisfied the following criteria were included in this meta-analysis (Table 1): soil runoff, water infiltration rate, soil biodiversity, nutrients recycling, mean yield, relative cost, constraints and adaptation to climate change. The aim of this study was not simply to include a large number of papers in the analysis, but rather to focus on the quality of those studies. So, the data from 43 studies in Sahelian countries were extracted and are analyzed in this paper (see Table 1). Through the meta-analysis, we would calculated the relative effects of each restoration action and for restoration overall on crop yield grain and coping to climate change.

**Table 1:** Adoption studies of traditional soil restoration strategies and binary code of the parameters of each practice.

Param	runoff	biodiversity	Soil nutrients cycling	water Infiltration	Relative cost	Labour	Mean yield	Adapation to climate change
Strategies								
Mulch	0 [12]	1 [13]	1 [14]	1 [14]	0 [15]	1 [16]	1 [17]	1 [18]
Half moon	1 [19, 20]	0 [21]	0 [19]	0 [21]	0 [22]	0 [22]	0 [19]	0 [23]
Stones line	1 [24]	0 [15]	0 [24]	1 [25]	0 [26]	0 [27]	0 [15]	0 [25]
Zaï	1 [28]	1 [29]	1 [29]	1 [29]	0 [21]	0 [26]	0 [30]	0 [31]
Zaï + mulch	1 [32]	1 [32]	1 [32]	1 [32]	0 [33]	0 [33]	1 [33]	1 [34]
Fallow	0 [35]	0 [36]	1 [35]	1 [35]	1 [37]	1 [37]	1 [35]	0 [38]
Parcage system (coralling)	0 [39]	1 [40]	1 [41]	0 [42]	1 [43]	0 [43]	1 [41]	1 [44]
Stones + Zaï	1 [45]	0 [46]	1 [45]	1 [45]	0 [47]	0 [47]	1 [45]	1 [21]
Crop rotation	0 [48]	0 [49]	1 [48]	0 [50]	1 [51]	1 [51]	1 [48]	1 [50]
Agroforestery	0 [52, 53]	0 [53, 54]	1 [53, 55, 56]	0 [53, 57]	1 [58, 59]	1 [59]	1 [53, 55]	1 [53, 56]
zero tillage	1 [60, 61]	0 [62, 63]	0 [60, 62, 64]	1 [60]	1 [61]	1 [61]	0 [62, 64]	0 [65]
Minimum tillage	1 [66]	0 [67]	0 [68]	0 [69]	0 [70]	0 [70]	0 [66]	0 [69]

[ …]: the reference number; 0 and 1 are codes respectively attributed to the lowest and the highest score attributed to the efficiency of a practice on a dependent variable.

#### 2.2.2 Analysis procedures

To evaluate the effects, relative variable importance was determined by setting the variable with the highest importance to 1 and calculating traditional soil strategies scores as a percentage of the most important variable. The independent variables that are likely to influence the mean yield and the adaptation to climate change are defined as soil runoff mitigation, soil biodiversity, soil nutrients recycling, water infiltration, labor, and relative cost. We built a database in which rows contained strategies and columns contained the variables of those agricultural practices.

### 2.3 Assessment of soil restoration strategies by farmers in response to climate change

A next step is through field surveys for interacting with farmers throughout 9 focus group discussions involving 102 participants with long-standing experience of local agriculture and climate to ensure a common opinion. We checked or verified the results of scholars with the perceptions of farmers about their preferences of traditional soil restoration practices to get more insights to the determination of the best practice. With this aim, we brought the same parameters previously used with scholars to understand farmer behavior. This focus group generated data on their determination to practice, to improve and take up the implementation of traditional soil restoration techniques. Each farmer scored the asset of the traditional soil restoration approaches performed in their field based on their consequences on the productivity as a weighting factor (rating from 1 to 5 with 1 being least important and 5 as the most important) where a high value represents elevated compliance. Farmers' probably uptake the traditional soil restoration technique ascertained by asking relevant questions on their willingness to continue practicing them in the future and increment the field acreage under the strategies. To be eligible for interview, farmers must have agriculture activity as the main occupation with at least 30 years of farming experience in order to give a clear trend in soil restoration success story. Collected information was grouped in two categories characteristic of respondents; information on knowledge (memories and experiences linked to soil degradation and climate changes) and information on farmers' behaviors.

### 2.4 Simulation of the effectiveness of zaï + mulching under climate change scenarios

Then, AquaCrop (version 6.1), a driven crop model to simulate yield response to water of several herbaceous crops, was used. It can alson be used for planning and decision making for production of under-utilised crops [71]. The AquaCrop model aims to balance simplicity, accuracy, robustness and user friendliness, and has the ability to operate on a minimum number of crop parameters [71]. The purpose of this simulation is to link both scholars and farmers views to climate change scenarios by assessing the effectiveness of zaï + mulching under climate change scenarios.

This practice is a combination of pit falls and mulch, adopted in order to reverse current processes of land degradation in aggradation.

AquaCrop 6.1 was selected for understanding crop responses to environmental change in one hand, covering most of the agricultural practices currently in use in the study region and for studying the impact of climate change on crop productivity (for example by running AquaCrop with both historical and future weather conditions) [72].

The model was calibrated using field trial data and from standard data (Table 2). By assuming the level of available soil water at planting, this allowed us to provide certain values for the millet variety description to forecast yield cumulative. Soil files were based on measurements.

**Table 2:** Summary of parameters used in the simulation.

Parameters	Definition	Way of Determination^a^
Sowing Date	08 June	F
Crop type	millet	F
Variety sown	HPK	F
Sowing density	10000 plants/ha	F
Soil evaporation	mm/day	F
Crop evapotranspiration	mm/day	F
Relative humidity	%	F
Source of weather data	Nasa	F
Soil type	Clay Loam	F
Maximum rooting depth	80 cm	F
Date of last rainfall entry	09 Sep	F
Harvest index	%	E
Initial canopy cover	%	E

a F= Field observed/measured data; E = calibrated.

### 2.5 Data analysis

The binary code was used to classify the variables of each practice presented in Table 1. One (1) was used to designate a high impact and zero (0) to denote a low impact of each practice on the variables. The outstanding advantage of this model is it capacity to analyze decisions and determine the effectiveness of a particular restoration strategy. The analysis is conducted using the data analysis package STATA 13.1. Coefficients in Probit model cannot be directly interpreted, we looked at the marginal effects which provide us a unified and intuitive way of describing relationship estimated with regression. Only positive marginal effect are retained and the practice with the high positive marginal effect is the best one.

Survey data is firstly analyzed by using summary statistics and frequency tests to identify smallholders choice of soil parameters-related issues, like the impact of soil restoration on soil nutrients, soil biodiversity, water infiltration and crop productivity and the farmers' own current adaptation measures. Next, the information are analyzed by using Probit models to check the conceptual links between, beliefs and experience of climate change and restoration strategies.

Downscaled historical climate data for the time 1980–2010, were performed as baseline conditions. As for projections, we used downscaling climate data for the scenario RCP 8.5 (representative concentration pathways) which correspond to radiative forcing of 8.5 W m^−2^.

This RCP 8.5 is the most pessimistic and results in a global average warming at the end of the 21st century of about 4 °C. Within this scenario, a global population increase drives a strong increase in croplands and pasture lands, especially in developing countries [73].

Daily climate data including maximum and minimum temperature, rainfall, solar radiation, maximum and minimum relative humidity, and evapotranspiration at a spatial resolution of 4 km × 4 km have been taken on giovanni.gsfc.nasa.gov. The latter site was used to extract soil information required by AquaCrop. Soil data was taking in the grid containing the study area, the predominant soil was chosen as input to the model. The outcome is given for two periods (2050 and 2100).

## 3 Results and discussion

### 3.1 Overview of traditional soil restoration practices studies

Table 1 presents the traditional soil restoration technologies used in this study and describes how each was coded in the analysis, as well as the effect of determinants on some soil parameters of agricultural land. Results from the Probit model show very strong positive association between zaï + mulch (Table 3). This practice has the highest regression coefficient of 10% affecting more the mean yield (*meanyield*) than the other practices. In addition to this, high coefficient of 22.22% of adaptation to climate change (*adaptclimatechang*) (Table 3). It means from our sample that zaï + mulch may compete more or is the most effective practice with the largest positive effects on the parameters compared to the other strategies in term of productivity and adaptation to climate change.

**Table 3:** Marginal effects after probit of *meanyield zaimulch and of adap.climchan zaimulch*.

Variables	Probit	$x\beta +\varepsilon \left(\frac{dy}{dx}\right)$
*meanyield zaimulch*	0.25	0.10
*Adap.climchan zaimulch*	*0.57*	*0.222*

Note: Explanatory variable is set equal to its median in the sample; N = 12; Log likelihood = -8.116 and 8.092.dy/dx are for discrete change of dummy variables from 0 to 1.

### 3.2 Perception of farmers

**Figure 1:**
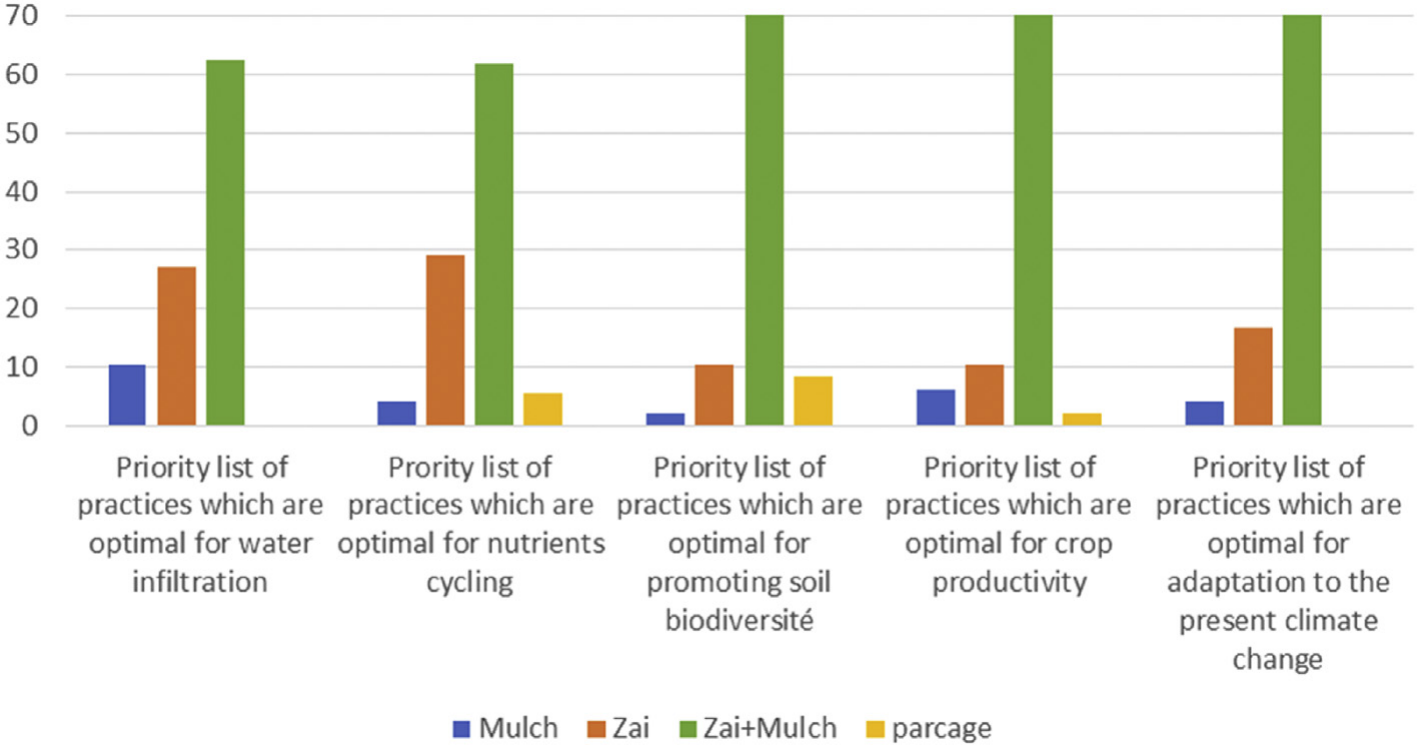
Specific adoption rates of traditional soil restoration practices.

First and foremost, farmers reported that doing Zaï and leaving high levels of crop residues behind, soil runoff is almost eliminated through this practice. Moreover, the use of crop residues in zaï + mulching also considerably increases water infiltration and therefore retention (i.e. less evaporation) by the soil. This means that there is less runoff of water, as well as a reduction in the amount of necessary watering requisite for millet. They also noticed that mulching is optimal for promoting soil fauna. The results of the farmers' survey revealed that among traditional soil restoration, under semi-arid conditions, zaï + mulching is a better practice that not only positively affect yields but it also allows to adaptation to the present climate change [74]. The consequences were found to have a positive effect on the adoption of zaï + mulch technics singularly. It has relatively higher rates of adoption (Figure 1). Table 4 relates the relationship between climate change awareness and the traditional soil restoration choice.

**Table 4:** Logistic regression between climate change awareness and the traditional soil restoration choice.

Variables	coef	Odds Ratio	std.Err	z	P>/|z|
*Do_you_believe_change_ex*	0.31	19.2	21.87	2.59	0.009

*Note: N* = 49; Log-Likelihood = -15.066.

### 3.3 Current and future yield simulations under association zaï + mulch

**Figure 2:**
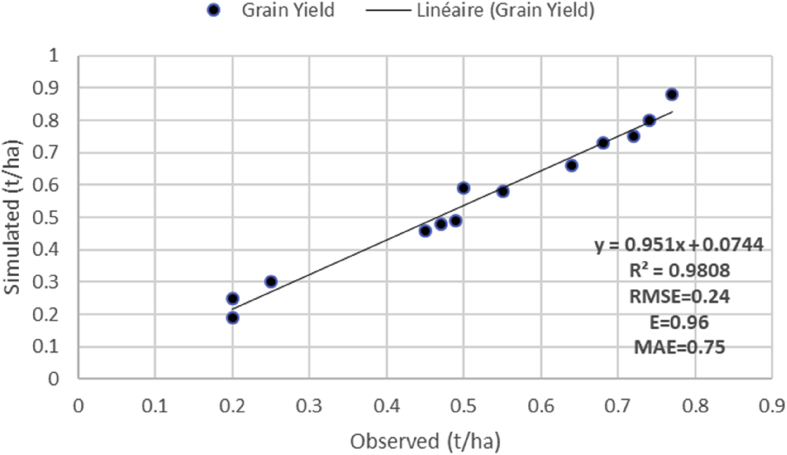
Model validation results in simulating and observed of pearl millet under rainfed treatment of the 2017–2018 season under climate change RCP 8.5. *R*^2^, coefficient of determination; RMSE, Root-mean-square error; E, model efficiency; MAE, mean absolute Error.

**Figure 3:**
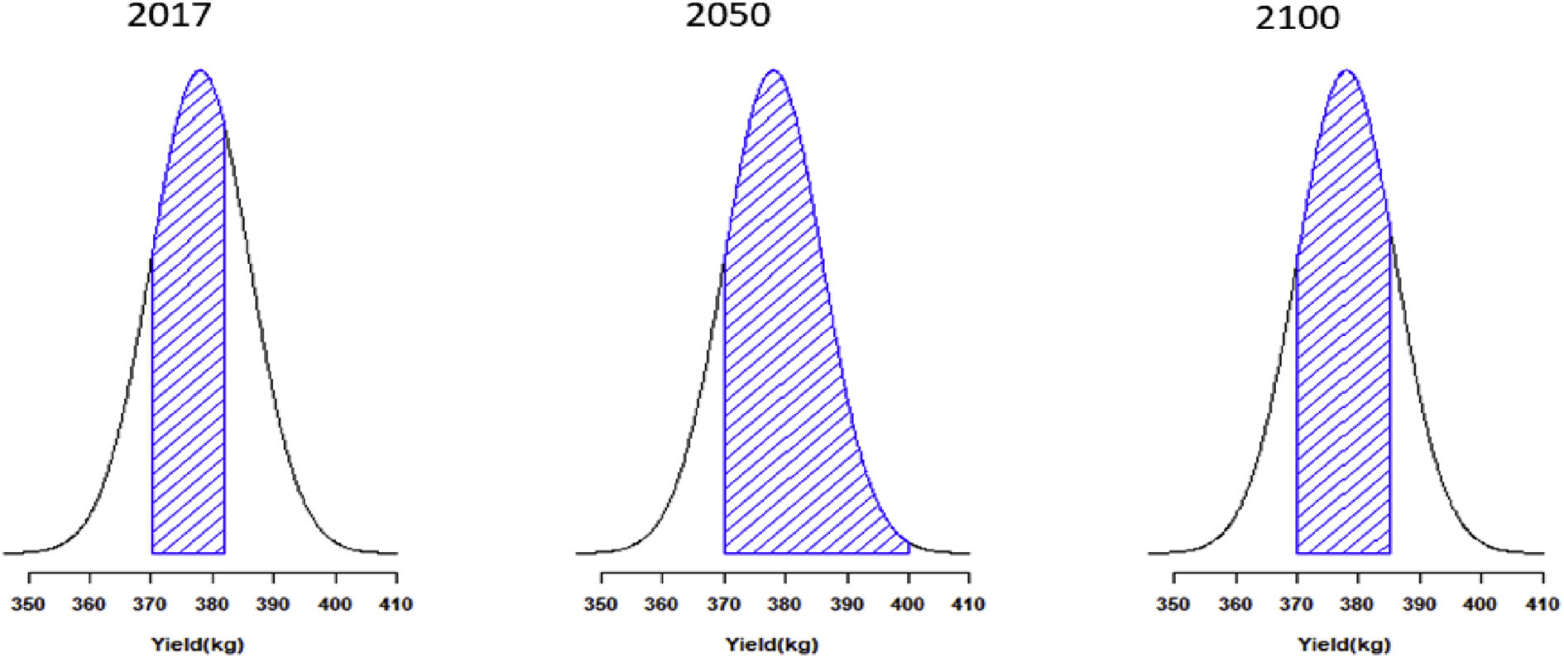
Millet grain yield simulated by AquaCrop under Climate Change Scenario RCP 8.5.

After calibration, the model reached the coefficient of efficiency (*R*^*2*^) level of 0.98. There is a good fit of measured and simulated values, as indicated by the low RMSE (0.24) and high MAE (0.75) and E (0.96) (Figure 2). A strong correlation between simulated and measured values was observed for the calibrated dataset (Y observed = 0.53 and Y measured = 0.68). Regarding climate change impacts, the model showed a positive impact to millet yield with an increase of 2.2% in 2050 compared to period of 2017 and 2100 (Figure 3), which may be attributed to more rainfall in 2050 and 2100 and increases in [75]. Compared to the baseline data, crop yield by the 2050 is projected to greatly raise 2 times than in control plots in RCP 8.5.

## 4 Discussion

From our sample of traditional soil restoration strategies, zaï + mulch may compete more or is the most effective practice with the largest positive effects on the parameters compared to the other strategies in term of productivity and adaptation to climate change. This can be attributed to the combination of multiple factors like increased of soil nutrients, enhanced water infiltration, higher soil carbon and macro-faunal activity leading to better soil structure that lowering soil temperature. The result supports the establishment of the relationship between climate change and mean yield and suggests the promotion of zaï + mulch practice in soil restoration that could be expected to affect the population of Southwest of Niger in a variety of ways. With regard to the performance of this practice, several authors, such as [29, 70], found that zaï + mulch is becoming more attractive because it minimized year to year yield variation (maximizes yield stability) under a changing climate. This could be explained by the fact that it maintains soil temperature lower. Another study conducted by [77] also relied largely on expert opinion, promote this practice as a restoration method on both soil productivity and climate change adaptation but was limited by financial means.

This result from farmers views on the association zai + mulch is consistent with the argument of several authors such as [33, 45, 78]. Accordingly, this practice is the most prevalent practice in the south western of Niger. The farmers' choice and willingness were informed by their hands-on experience given that they had tested the techniques on the farms and practically observed the benefits.

Results (Table 4) revealed that awareness on climate change shows significant association between information receipt and traditional soil restoration methods. This allow them to adopt an effectiveness traditional soil restoration practice in order to alleviate water shortages, worsening soil conditions, and other negative effects of climate change (Table 4). However, as stated by [52], there is still a need to raise more climate change awareness among farmers so as to increase traditional soil restoration practices.

Yields obtained by 2050 are consistently higher (ranging from 870 to 1590 kg/ha) than those obtained in control plots in RCP 8.5 (average 55–90 kg/ha). Through the current based study, yields of millet may significantly profit are expected to take advantage from climate changes in this 21^st^ century under RCP 8.5 [79]. Despite the fact that the response of crop growth to future temperature anomalies looks even more uncertain [80], the observed temperature in the southwest of Niger and an increase before crop maturation under future conditions will mitigate effects of temperature on yield production. The positive consequences of a higher concentration of atmospheric CO_2_, anyhow, is clearer.

## 5 Conclusion

Zaï + mulch can be on the one hand a promising approach toward climate change adaptation while sustaining food security in southwest of Niger. On the other hand the strategy potentially offered good potential to increase productivity by 2 times higher than in control plots. Requiring applications of crop residues, manure and labors, it could be expensive in terms of cost effectiveness for farmers. The strategy increases millet production under a changing climate even if chance of crop failure can occur due to precipitation variability. The developed model AquaCrop has been a useful tool to analyze the merits of association *zaï* + mulch practice by indicating that this practice may be considered among the most important practice to restore a degraded soil. AquaCrop model recommends that millet production can be continued in the study area under climate change conditions in the future.

Some estimations based on findings corroborate that mulch provide almost 0.05 m of added water to crops, then zaï + mulching has a water penetration. This makes the practice an excellent opportunity to drought affected areas such as the Southwest of Niger.

Association zaï + mulch could be considered as the best practice that can participate to a successful adaptation to reduce risk from climate change at the same time by reducing the vulnerability of farmers in Southwest of Niger.

## Declarations

### Author contribution statement

A. Amadou Issofou: Conceived and designed the analysis; Performed the experiments; Analyzed and interpreted the data; Contributed reagents, materials, analysis tools or data; Wrote the paper.

S. Konaté: Conceived and designed the analysis; Wrote the paper.

I. Soumana: Performed the experiments; Analyzed and interpreted the data; Contributed reagents, materials, analysis tools or data; Wrote the paper.

G. Maman: Performed the experiments; Analyzed and interpreted the data; Contributed reagents, materials, analysis tools or data; Wrote the paper.

A. Mahamane: Conceived and designed the experiments; Wrote the paper.

### Funding statement

This work was supported by WASCAL–West African Science Service Center on Climate change and Adapted Land use–02CCBI16.

### Competing interest statement

The authors declare no conflict of interest.

### Additional information

No additional information is available for this paper.
